# Subacute combined degeneration of the spinal cord with concomitant autoimmune disease: report of 2 cases

**DOI:** 10.1590/1414-431X2021e11355

**Published:** 2021-07-16

**Authors:** Tian-Fang Jiang, Jia Zheng, Xu Chen

**Affiliations:** 1 Department of Neurology, Shanghai Eighth People's Hospital Affiliated to Jiang Su University, Shanghai, China

**Keywords:** Subacute combined degeneration of spinal cord, Vitamin B12, Anti-intrinsic factor antibody, Autoimmune disease, Case report

## Abstract

The etiology of subacute combined degeneration (SCD) of the spinal cord is closely associated with vitamin B12 (VitB12) deficiency. The clinical manifestations of SCD are complex and vary substantially. Due to some SCD patients with atypical manifestations and concomitant autoimmune disorders, the probability of misdiagnosis and missed diagnosis is still relatively high in the early stage. We report the cases of two patients who were missed or misdiagnosed at another hospital because of the normal initial VitB12 level and partial overlap of clinical manifestations, finally diagnosed as SCD with atypical manifestations and concomitant autoimmune disorders, pharyngeal-cervical-brachial Guillain-Barre syndrome in Case 1 and SCD with autoimmune thyroiditis in Case 2. After undergoing corresponding treatment, death was reported in Case 1 and improvement in Case 2. Analysis of the clinical manifestations and investigation of the underlying pathogenesis in such patients could help improve the rate of early diagnosis and allow timely treatment of SCD, thereby preventing disease progression and poor clinical outcomes.

## Introduction

Subacute combined degeneration (SCD) of the spinal cord is a rare disease characterized by demyelination of dorsal and lateral columns due to vitamin B12 (VitB12) deficiency. SCD with comorbidities such as autoimmune diseases is less reported, of which concomitant presence increases the difficulty in diagnosis. Here, we report two cases of SCD accompanied by autoimmune diseases.

## Case 1

A 59-year-old man was admitted to the Department of Neurology of the Shanghai Eighth People's Hospital in November 2019 due to a 1-month history of repeated falls and urination/defecation disorders. In September 2019, he was diagnosed with lumbar disc protrusion at another hospital owing to progressive bilateral lower-limb numbness and unsteady walking for 1 year and waist pain for 6 months. His serum VitB12 level was normal and he underwent minimally invasive surgery (percutaneous endoscopic interlaminar lumbar discectomy) without improvement at the time. He had a 30-year history of drinking (yellow rice wine, ∼250 g/d), and denied a history of nitrous oxide abuse or anesthesia.

A physical examination at our hospital showed that the patient had a clear mind and multiple sites of pallor in the face, sclera, and lips. Muscle tension and strength were normal in the upper bilateral limbs and decreased in the bilateral lower limbs (left, grade III+; right, grade III+ [proximal] to grade II [distal]). Tendon reflexes were present (++) in bilateral upper limbs and absent in bilateral lower limbs. Pinprick sensation was decreased below the T6 level; vibration, motor, and position sensations were decreased in both lower limbs. The Babinski and Chaddock signs were positive on the left side. Blood tests showed: red blood cells (RBCs), 1.93×10^12^/L (normal range (NR) 4.3-5.8*10^12^/L); hemoglobin (Hb), 82 g/L (NR 130-175 g/L); hematocrit (HCT), 23.2% (NR 37-50%); mean corpuscular volume (MCV), 114.9 fL (NR 82-100 fL); mean corpuscular hemoglobin (MCH), 42.5 pg (NR 27-34 pg); red blood cell distribution width (RDW), 62.9% (NR 11.9-14.5%); VitB12, 30 pM (NR 138-652 pM); folic acid (FA), 16.11 nM (NR 7.0-46.4 nM); homocysteine (HCY), 118 μM (NR 5-15 nM); and serum copper, 13.56 μM (NR 7.12-21.29 μM). Anti-nuclear antibody (ANA), anti-cardiolipin antibody (ACA), anti-neutrophil cytoplasmic antibody (ANCA), rheumatoid factor (RF), syphilis, HIV, and tumor markers were negative. Lumbar puncture and cerebrospinal fluid (CSF) examination yielded normal results; autoantibody and oligoclonal bands were negative.

**Figure 1:**
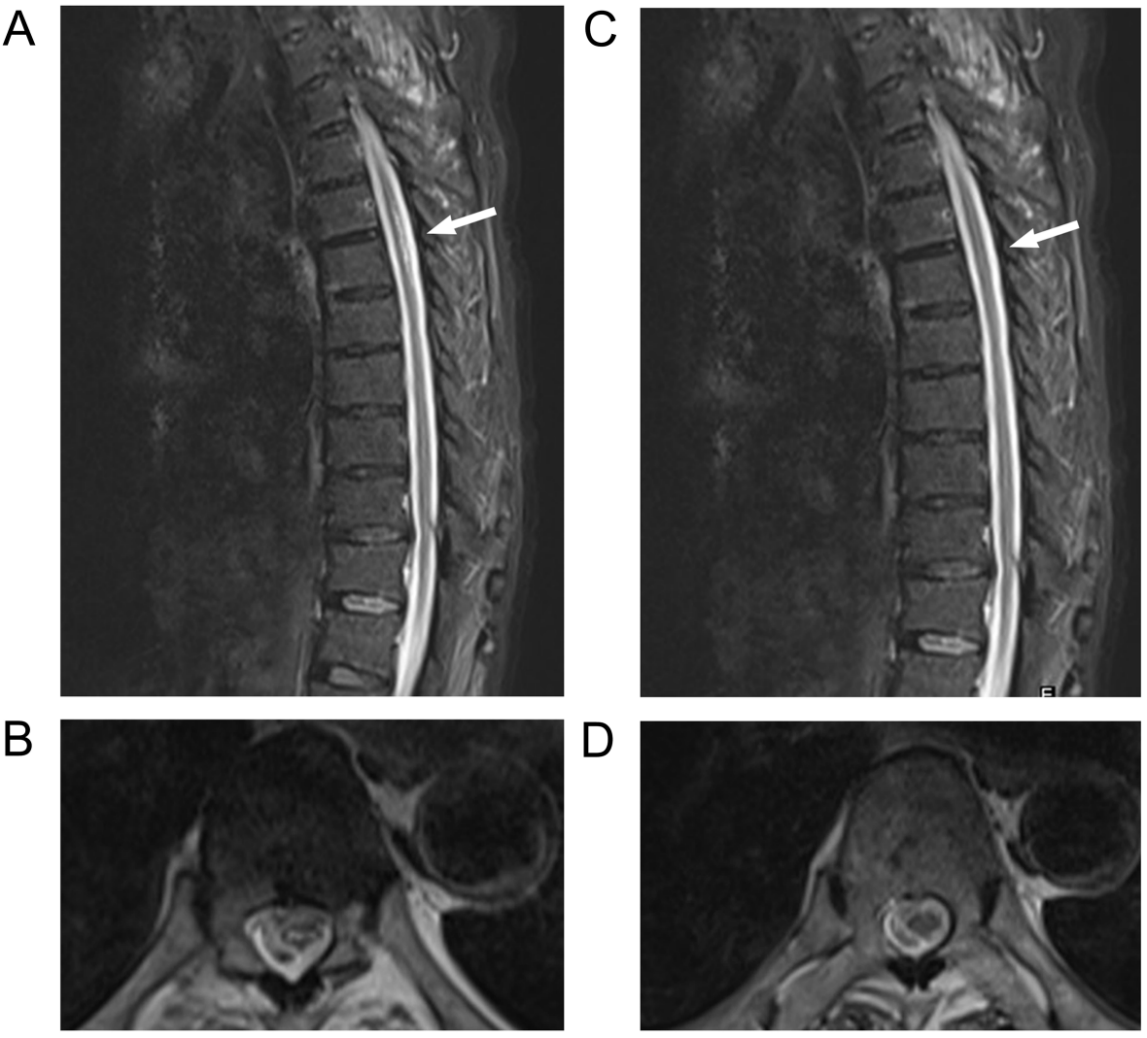
T_2_-weighted magnetic resonance images of thoracic vertebra in Case 1 at the time of the first (**A** and **B**) and last admissions (**C** and **D**). **A**, Sagittal view shows the thoracic spinal cord with a high-signal focus (arrow). **B**, Axial view shows the thoracic spinal cord and the “small print” sign (at the T5-T6 level; arrow). **C**, Sagittal and **D**, axial views show that the thoracic spinal cord lesions had partially recovered (at the T5-T6 level; arrows).

Electromyography showed reduced conduction velocity of multiple sensory and motor nerves in both lower limbs; no evoked potentials were induced, and P40 of the left tibial nerve was not induced. Thoracic vertebral magnetic resonance image (MRI) showed long T2 signals in both dorsal and lateral columns at T5-T6, i.e., the “small character” sign (Figure 1A and B). Cervical vertebral MRI showed C3-C4 intervertebral disc protrusion and C4-5, C5-6, and C6-7 intervertebral disc bulging.

The patient was diagnosed with SCD, megaloblastic anemia, and cervical and L5-S1 lumbar intervertebral disc protrusion. The patient gradually stopped drinking after hospitalization and received daily intramuscular injections of mecobalamine (1 mg; Eisai Pharmaceutical Co., Ltd, China) and oral folic acid (FA; Tianjin Lisheng Pharmaceutical Co., Ltd, China), VitB12 (Eisai Pharmaceutical Co., Ltd, China), and VitB6 (CR Double-Crane Pharmaceutical Co., Ltd, China). Following treatment, the symptoms improved and all biochemical examination results returned to normal. Intramuscular mecobalamine was administered every other day for 4 weeks and was gradually replaced with oral mecobalamine. The patient could walk with assistance and urinate/defecate normally.

**Figure 2:**
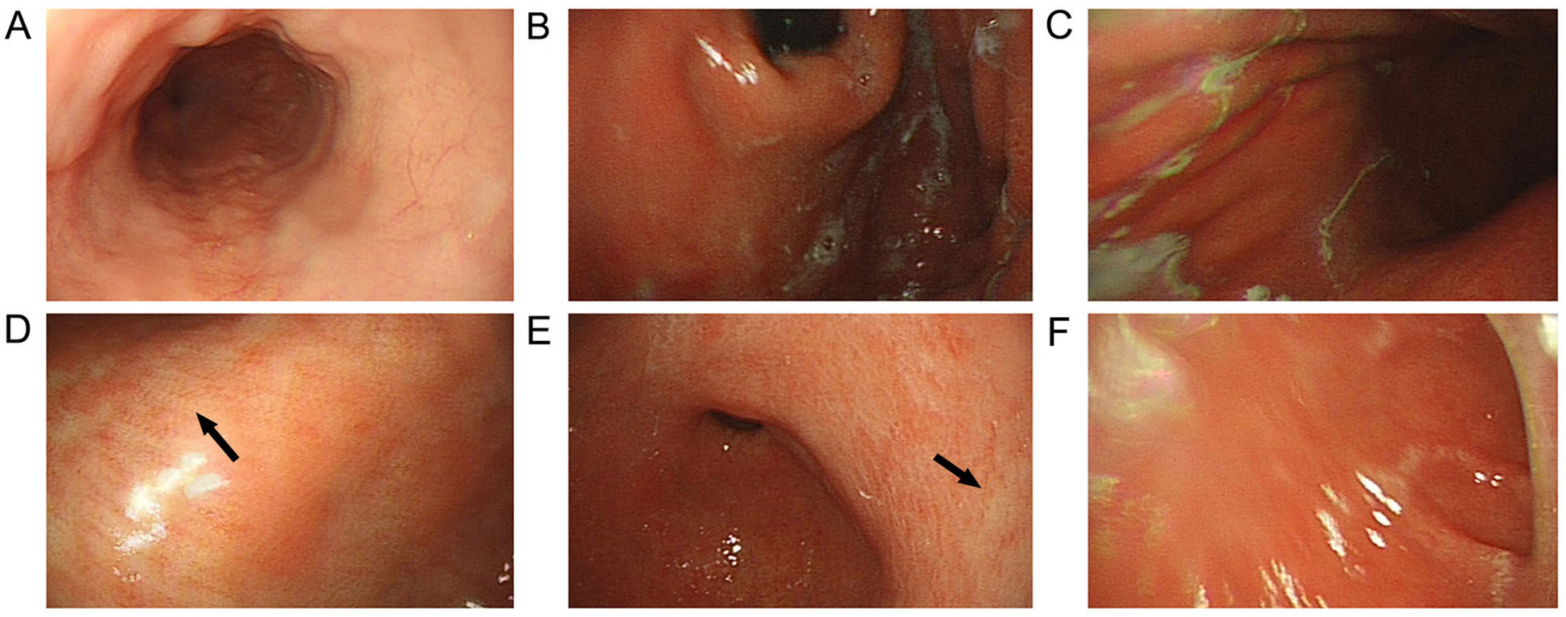
Gastroscopy images of chronic atrophic gastritis in Case 1. **A**, esophagus; **B**, gastric fundus; **C**, gastric body; **D**, gastric angular incisure; **E**, gastric antrum; **F**, duodenal bulb. The gastric mucosa at the atrophic site is red and white (arrows).

**Figure 3:**
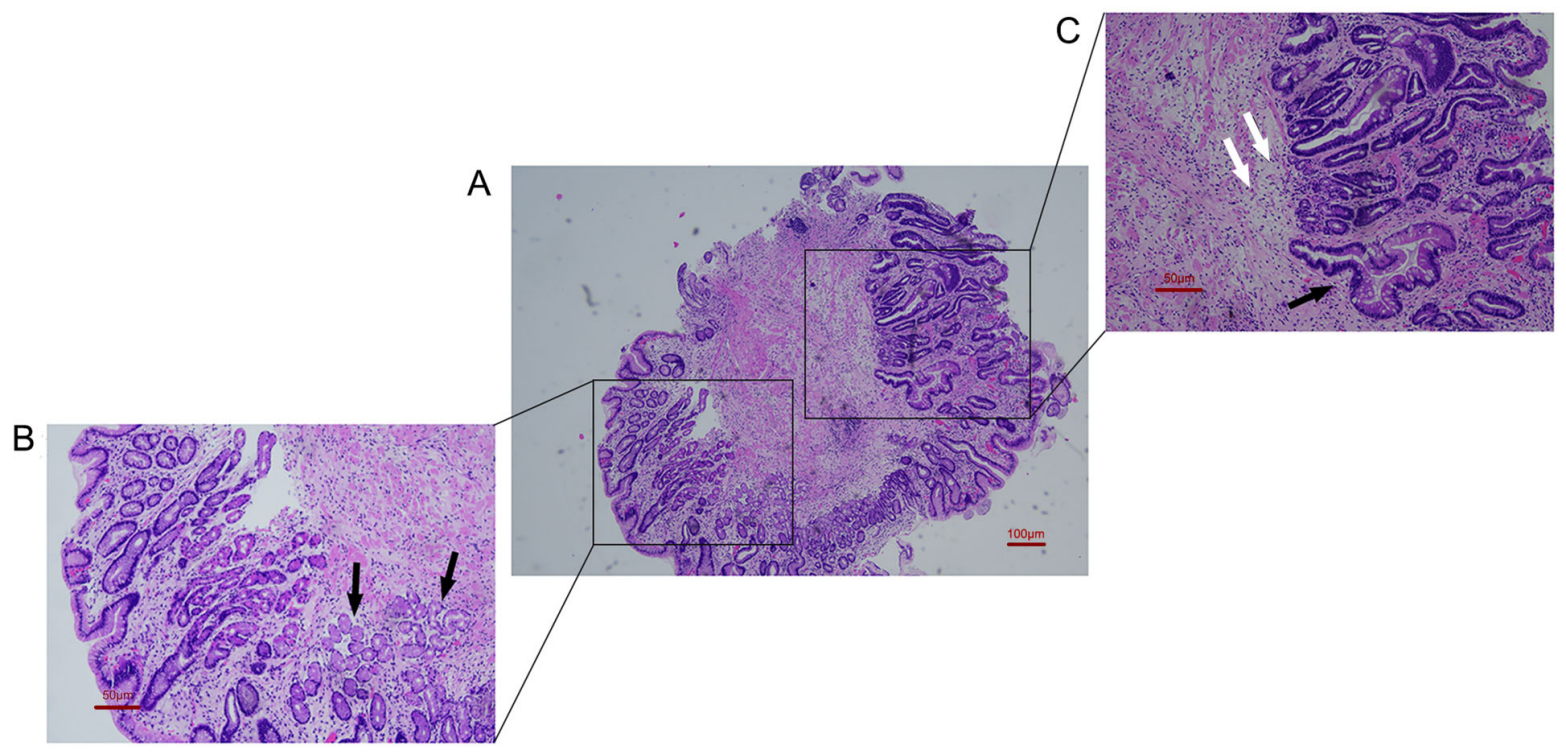
Pathological examination of a gastric antrum specimen in Case 1. **A**, Overall view (×40, scale bar 100 μm). **B**, Local view (×100, scale bar 50 μm) showing a small amount of normal antral glands (black arrows). **C**, Local view (×100, scale bar 50 μm) showing that the normal glands in the submucosal basal layer are completely atrophied (white arrows) and replaced with numerous intestinal goblet cells and metaplastic glands (black arrows).

However, the patient was re-admitted to our department 1 month later due to worsening lower-limb weakness for 1 week. Re-examination showed VitB12 >1000 pM and positive serum anti-intrinsic factor antibody. Gastroscopy and biopsy showed type A chronic atrophic gastritis (Figures 2 and 3). Intramuscular mecobalamine (1 mg/injection, one injection every other day) was administered for 4 weeks. The lower-limb weakness improved. Pre-discharge examinations showed that deep sensations in the lower limbs had improved since the first hospitalization. Muscle strength also improved (left, grade III+; right, grade IV- [proximal] to III [distal]). Tendon reflexes were positive in both lower limbs, and urination/defecation was normal.

In 2020, the patient was repeatedly admitted to our department for worsening of lower-limb weakness, during which VitB12 treatment was continued, and VitB12 levels were maintained normal. In March 2020, the patient was treated in another hospital for bilateral upper-limb weakness, cough, and difficulty in chewing for 3 days. However, his symptoms did not improve after intramuscular mecobalamine, citicoline sodium infusion, and oral VitB and FA.

After 4 days, he was admitted to our department. Before disease onset, he had diarrhea, which improved after 2 days of oral berberine. Physical examination showed that the patient had a clear mind, but a tender neck, feebleness, hoarseness during speaking, and difficulty in chewing, coughing, shrugging, and raising his head. The lifting of the soft palate was bilaterally poor, and the uvula was in the middle. Both upper-limb muscle tension and strength were reduced (proximal, grade IV-; distal, grade III), and tendon reflexes were positive. Lower-limb muscle tension and strength were identical to the previous examination. No pathological signs were found. Routine blood tests showed normal results. Stool culture 7 days after onset showed *Campylobacter jejuni* infection, which was treated with gentamicin. Electromyography 9 days after onset showed increased motor latency and decreased amplitude of the terminal median nerve. Reduced motor-nerve conduction velocity, decreased amplitude, and delayed F wave were seen. Increased motor latency of the terminal ulnar nerve, with normal conduction velocity, was found. Fibrillation potentials were detected in the distal upper limbs. No abnormalities were found after repeated electrical stimulations.

Cranial MRI showed no abnormalities. Cervical and thoracic vertebral MRI showed similar changes in the cervical vertebrae as in the previous examinations, along with T10-11 intervertebral disc bulging, thoracic vertebral degeneration, and partial recovery of the T5-T6 spinal cord lesion (Figure 1C and D). Lumbar puncture 11 days after onset showed: pressure, 150 mmH_2_O; white blood cells, 1×10^6^/L; and total protein, 488 mg/L. Blood GQ1b and GT1a antibodies were positive, while GM1, GM2, GM3, GT1b, GD1a, and GD1b antibodies were negative. Paraneoplastic antibodies (Hu/Yo/Ri), CSF oligoclones, and AQP4 were negative.

The patient was diagnosed with pharyngeal-cervical-brachial (PCB) Guillain-Barre syndrome (GBS) in SCD. Intravenous γ-globulin (0.4 g/kg) was administered for 5 days beginning 9 days after onset, and on day 12, the following changes were seen: sudden dyspnea with repeated diarrhea; finger oxygen desaturation index, 93%; heart rate, 110 bpm; decreased consciousness; and moderate coma. Oral tracheal intubation and mechanical ventilation were performed immediately. In addition, 1 g methylprednisolone was injected intravenously for 3 days, and gradually tapered until discontinuation. By day 20, the patient could open his eyes when called, had bilaterally reduced light reflexes, and stable vital signs. Anti-infection and supportive treatments were continued, with little effect. Due to the heavy financial burden, his family voluntarily gave up the treatment, and the patient passed away.

## Case 2

A 70-year-old woman was admitted to our department with bilateral lower-limb numbness and unsteady walking for 1 month. In January 2020, she was diagnosed with anxiety disorder at another hospital, due to loss of appetite and bilateral lower-limb numbness for 3 months. She received Deanxit treatment, without improvement.

At our hospital in February 2020, she reported a 1-month history of weight loss, diarrhea alternating with constipation, and multiple episodes (>10/d) of irradiative, electric shock-like pain in both lower limbs, which lasted for several seconds and then subsided. She denied a history of nitrous oxide abuse or anesthesia.

Physical examination showed normal consciousness, slow reactions, low emotions, and skin desquamation at multiple sites of the hands and feet. We found normal muscle tension and slightly reduced muscle strength in the limbs (upper, grade V-; lower, grade IV-), with positive tendon reflexes (upper limbs, ++; lower limbs, +++). Pinprick and vibration sensations were decreased in both lower limbs. The Babinski sign was detected in both lower limbs; the Chaddock sign was present in the left lower limb. The Romberg sign was positive. Blood tests showed the following: RBCs, 2.7×10^12^/L (NR 4.3-5.8*10^12^/L); Hb, 81 g/L (NR 130-175 g/L); HCT, 29.90% (NR 37-50%); MCV, 114.5 fL (NR 82-100 fL); MCH, 35.2 pg (NR 27-34 pg); RDW, 69.4% (NR 11.9-14.5%); VitB12, 61 pM (NR 138-652 pM); FA, 45.3 nM (NR 7.0-46.4 nM); serum copper, 16.31 μM (NR 7.12-21.29 μM); serum anti-intrinsic factor antibody, positive; HCY, normal; ANA, ACA, ANCA, and RF, negative; syphilis and HIV, negative.

**Figure 4:**
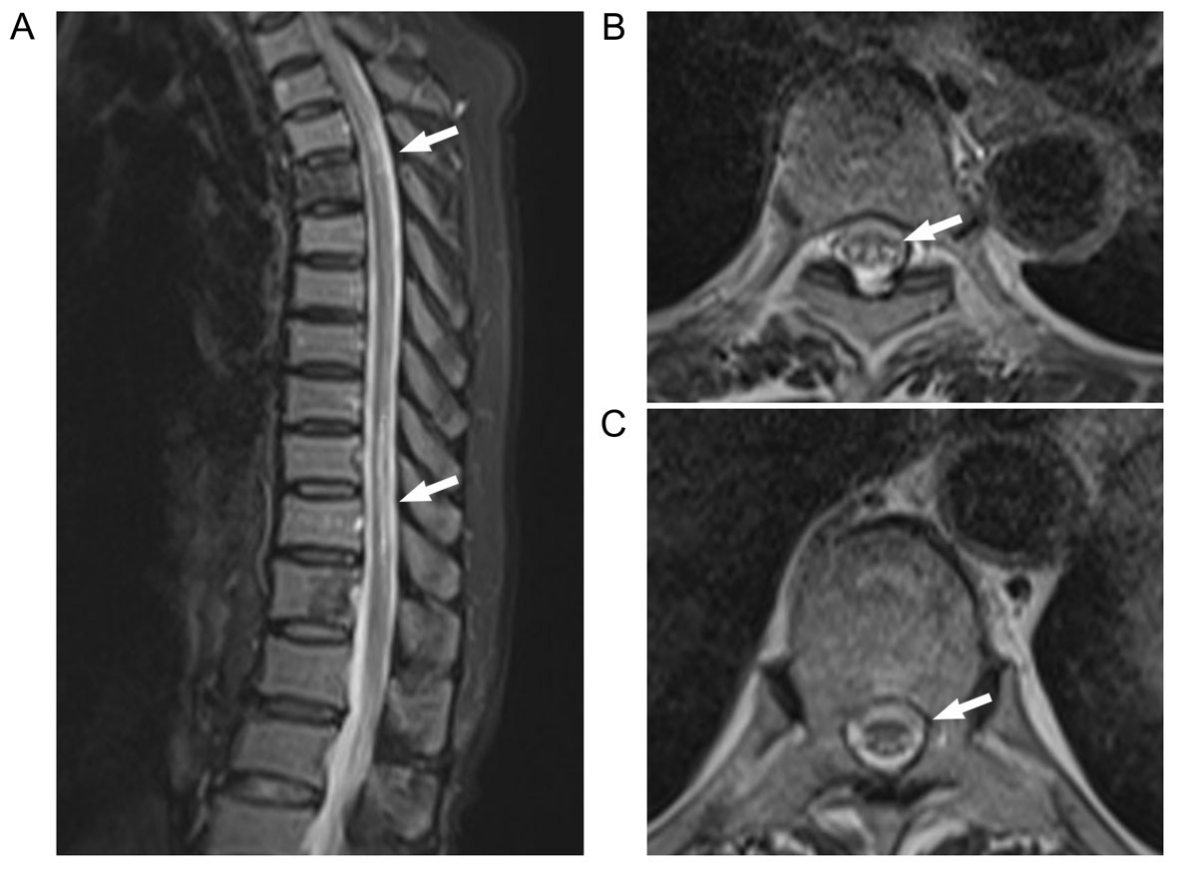
T_2_-weighted magnetic resonance images of thoracic vertebra in Case 2. **A**, Sagittal view shows high-signal intensity lesions in the thoracic spinal cord (arrows). **B** and **C**, Axial views show the spinal cord “small print” sign at different positions in the thoracic segment (**B**, T5 level; **C**, T10-T12 level; arrows).

Thoracic vertebral MRI showed long T1 and T2 signals in both dorsal and lateral columns of T5-T6, i.e., the “small character” sign (Figure 4). Electromyography showed substantially decreased amplitude and conduction velocity of the tested sensory and motor nerves, multiple peripheral nerve injuries, motor and sensory nerve axonal damage, and myelin injures. Thyroid tests showed the following: free thyronine, 4.0 pM (NR 3.1-6.8 pM); free thyroxine, 11.24 pM (NR 12-22 pM); thyroid-stimulating hormone, 5.97 μIU/mL (NR 0.27-4.2 μIU/mL), and thyroid peroxidase antibody, 93.96 IU/mL (NR 0-34 IU/mL). Thyroid ultrasonography showed bilateral heterogeneous texture of the thyroid with a hypoechoic nodule. Fine needle aspiration cytology showed benign nodules.

The patient was diagnosed with SCD, malignant anemia, and autoimmune thyroiditis (AITD). Daily intramuscular injection of mecobalamine (1 mg/) and oral FA, VitB12, and VitB6 were administered for 2 weeks. The VitB12 level returned to normal. Lower-limb muscle strength (grade IV+ to V-) and deep sensation improved, tendon reflexes were bilaterally positive (++), and urination/defecation were normal. The patient could stand without help. She was referred to the Endocrine Department for AITD treatment.

## Discussion

We have reported two rare cases of SCD with atypical manifestations and complicated with autoimmune disorders (Supplementary Table S1). Wong summarized the diagnostic criteria for VitB12 deficiency (1). In short, the most frequent serum VitB12 cut-off to diagnose VitB12 deficiency is 150 pM (203 pg/mL). However, currently there is no accurate, or “gold standard” test for the diagnosis of VitB12 deficiency. Clinical diagnosis is usually based on reduced serum VitB12 level and effective VitB12 replacement therapy. The laboratory assessment includes serum VitB12 level, complete blood count, and blood film examination. Detection of homocysteine and methylmalonic acid in blood can improve the positive rate of diagnosis.

In Case 1, the normal initial VitB12 level led to initial misdiagnosis, which means VitB12 may not be a reliable marker for SCD (2,3). In addition, sensory and vesicorectal disturbances that we reported are rare in SCD. Puntambekar et al. (4) reported a case of SCD with similar manifestations. In addition, the disease was exacerbated after intramuscular VitB12 treatment was changed to oral treatment. This might be attributable to intestinal VitB12 malabsorption due to anti-intrinsic factor antibody and chronic atrophic gastritis. Thus, patients with anti-intrinsic factor antibody may require life-long intramuscular VitB12 treatment. It is worth noting that Case 1 had a history of cervical disc herniation. It has been reported that the reversible compression injury caused by SCD and degenerative cervical myelopathy requires sufficient vitamin B12 for spinal cord remyelination (5). The upper limbs of this patient were not involved in the first and second stage of the disease. In the third stage, the muscle strength of his upper limbs was decreased, however the acute onset and rapid progression of obvious symptoms and signs of polyneuropathy could not be attributed to cervical disc herniation.

Case 2 also had an atypical onset. Moreover, AITD was present as a comorbidity and manifested as hypothyroidism, which can induce emotional disturbance and gastrointestinal symptoms, and mask sensory manifestations in early SCD. Approximately 16% of AITD patients have concomitant malignant anemia, with parietal-cell damage, inhibition of gastric acid secretion, and gastric mucosal atrophy, leading to reduced VitB12 absorption (6). Furthermore, AITD-induced hypothyroidism can cause gastrointestinal dysfunction, abdominal distension, constipation, and reduced food intake, thereby worsening the VitB12 deficiency. Thus, AITD-induced malignant anemia and VitB12 deficiency could increase the difficulty in diagnosing SCD.

As shown in the two cases in this report, the role of autoimmune dysfunction in SCD has been largely overlooked (7). Neumann et al. (8) reported that SCD with malignant anemia was common among elderly patients. Several cases of SCD combined with autoimmune diseases, including rheumatoid arthritis (9), autoimmune gastritis (10), and ulcerative colitis (11), have been reported. The findings in our cases suggested that SCD-associated malignant anemia can only manifest as atypical gastrointestinal reactions or no reaction. Therefore, anti-intrinsic factor antibody testing and early gastroscopy are critical for such patients. Anti-intrinsic factor antibody is highly specific for SCD, but its sensitivity is limited, which could be influenced by serum VitB12 levels. Therefore, for cases of suspected SCD, anti-intrinsic factor antibodies should be measured at least 2 weeks before or after oral VitB12 supplementation (7,12).

PCB GBS in SCD is especially rare. Its clinical manifestations partially overlap with those of SCD and are easily misdiagnosed as SCD progression. In Case 1, electromyography and autoantibody tests revealed the correct diagnosis as severe PCB GBS (13). Among patients with PCB GBS, approximately 71% have antecedent upper-respiratory-tract infection, 30% have diarrhea, and 31% have serological evidence of *Campylobacter jejuni* infection (14). The lower extremities are usually not involved in PCB GBS (15). This patient had a prodromal intestinal infection. The typical manifestations are rapidly progressing oropharyngeal, cervical, and arm weakness, accompanied by weakened upper-extremity reflexes. Furthermore, positive anti-ganglioside antibodies and typical upper-extremity damage on electromyography indicated PCB GBS. In Case 1, lower-limb muscle strength and reflexes did not worsen compared with the previous hospitalization, which can be explained by partial SCD recovery. The patient tested positive for multiple autoantibodies, including anti-intrinsic factor, anti-GT1a, and anti-GQ1b antibodies, which led to the successive onset of central and peripheral demyelinating diseases.

The comorbidity of AITD as in Case 2 is also relatively rare. This patient had diarrhea alternating with constipation, malignant anemia, and weight loss. These symptoms may be attributable to SCD-induced autonomic nerve dysfunction and overgrowth of small intestinal bacteria due to hypothyroidism, autoimmune dysfunction, or immunosuppression (16). The above findings showed that autoimmune disorders can cause various comorbidities in SCD.

In conclusion, for SCD patients with concomitant autoimmune disorders, the probability of misdiagnosis and missed diagnosis is still high, and autoimmune mechanisms can play an important role in the pathogenesis of SCD. More studies are required to investigate the underlying mechanisms. In the reported cases, we considered VitB12 deficiency to be a “result” rather than a “cause”. Patients comorbid with multiple autoimmune diseases have immune disease diathesis, and they produce a variety of autoantibodies, including anti-ganglioside antibody, anti-thyroid autoantibody, anti-intrinsic factor antibody, and others. The anti-intrinsic factor in both of our two patients was positive, which resulted in the malabsorption and deficiency of VitB12. The mechanism of autoimmune diseases as comorbidities may be related to many genetic susceptibility genes, including human leukocyte antigens type 2 (17,18). Future studies are being designed to collect a large number of clinical data of these patients and conduct gene analysis, which will help to clarify the specific pathogenesis.

## Supplementary Material

Click here to view [pdf].
